# 5-[(1*R*,2*R*,4*R*)-2-Meth­oxy-1,7,7-tri­methylbi­cyclo­[2.2.1]hept-2-yl]-1*H*-tetra­zole

**DOI:** 10.1107/S1600536813014700

**Published:** 2013-06-08

**Authors:** Jan W. Bats, Peter Schell, Joachim W. Engels

**Affiliations:** a Institut für Organische Chemie und Chemische Biologie, Universität Frankfurt, Max-von-Laue-Strasse 7, D-60438 Frankfurt am Main, Germany

## Abstract

The title compound, C_12_H_20_N_4_O, undergoes a phase transition on cooling. The room-temperature structure is tetra­gonal (*P*4_3_2_1_2, *Z*′ = 1), with the meth­oxy­bornyl group being extremely disordered. Below 213 K the structure is ortho­rhom­bic (*P*2_1_2_1_2_1_, *Z*′ = 2), with ordered mol­ecules. The two independent mol­ecules (*A* and *B*) have very similar conformations; significant differences only occur for the torsion angles about the C_born­yl_—C_tetra­zole_ bonds. The independent mol­ecules are approximately related by the pseudo-symmetry relation: *x_B_* = −1/4 + *y_A_*, *y_B_* = 3/4 - *x_A_* and *z_B_* = 1/4 + *z_A_*. In the crystal, mol­ecules are connected by N—H⋯N hydrogen bonds between the tetra­zole groups, forming a pseudo-4_3_ helix parallel to the *c*-axis direction. The crystal studied was a merohedral twin with a refined twin fraction value of 0.231 (2).

## Related literature

For the chemical background and synthesis of the title compound, see: Schell & Engels (1997 ▶, 1998 ▶). For related structures, see: Ohno *et al.* (1999 ▶).
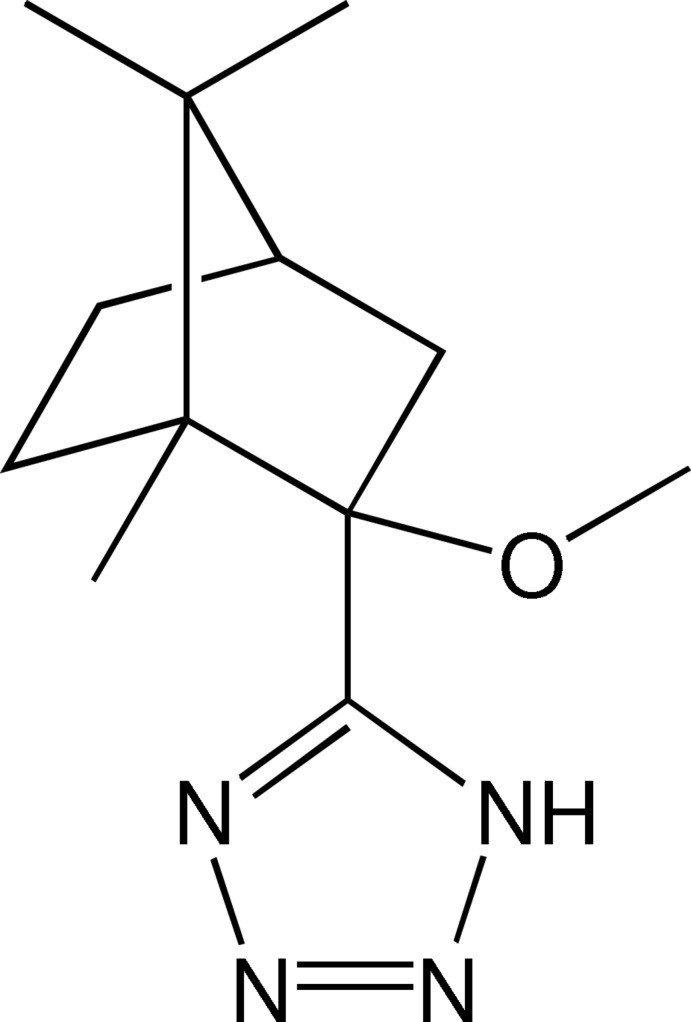


## Experimental

### 

#### Crystal data

C_12_H_20_N_4_O
*M* *_r_* = 236.32
Orthorhombic, 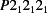

*a* = 13.298 (3) Å
*b* = 13.608 (4) Å
*c* = 14.356 (3) Å
*V* = 2597.9 (11) Å^3^
*Z* = 8
Mo *K*α radiation
μ = 0.08 mm^−1^
*T* = 194 K
0.65 × 0.24 × 0.20 mm

#### Data collection

Siemens SMART 1K CCD diffractometer
31860 measured reflections
3283 independent reflections
2259 reflections with *I* > 2σ(*I*)
*R*_int_ = 0.109

#### Refinement

*R*[*F* ^2^ > 2σ(*F* ^2^)] = 0.069
*wR*(*F* ^2^) = 0.138
*S* = 1.08
3283 reflections
322 parameters
2 restraints
H atoms treated by a mixture of independent and constrained refinement
Δρ_max_ = 0.20 e Å^−3^
Δρ_min_ = −0.21 e Å^−3^

### 

Data collection: *SMART* (Siemens, 1995 ▶); cell refinement: *SMART*; data reduction: *SAINT* (Siemens, 1995 ▶); program(s) used to solve structure: *SHELXS97* (Sheldrick, 2008 ▶); program(s) used to refine structure: *SHELXL97* (Sheldrick, 2008 ▶); molecular graphics: *SHELXTL* (Sheldrick, 2008 ▶); software used to prepare material for publication: *SHELXL97*.

## Supplementary Material

Crystal structure: contains datablock(s) global, I. DOI: 10.1107/S1600536813014700/su2603sup1.cif

Structure factors: contains datablock(s) I. DOI: 10.1107/S1600536813014700/su2603Isup2.hkl

Supplementary material file. DOI: 10.1107/S1600536813014700/su2603Isup3.cml

Additional supplementary materials:  crystallographic information; 3D view; checkCIF report

## Figures and Tables

**Table 1:** Hydrogen-bond geometry (Å, °)

*D*—H⋯*A*	*D*—H	H⋯*A*	*D*⋯*A*	*D*—H⋯*A*
N1—H1*A*⋯N7	0.88 (1)	2.09 (3)	2.850 (6)	144 (5)
N5—H5*C*⋯N3^i^	0.88 (1)	2.02 (2)	2.857 (6)	160 (5)

Symmetry code: (i) 
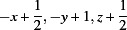
.

**Fig. 1.**
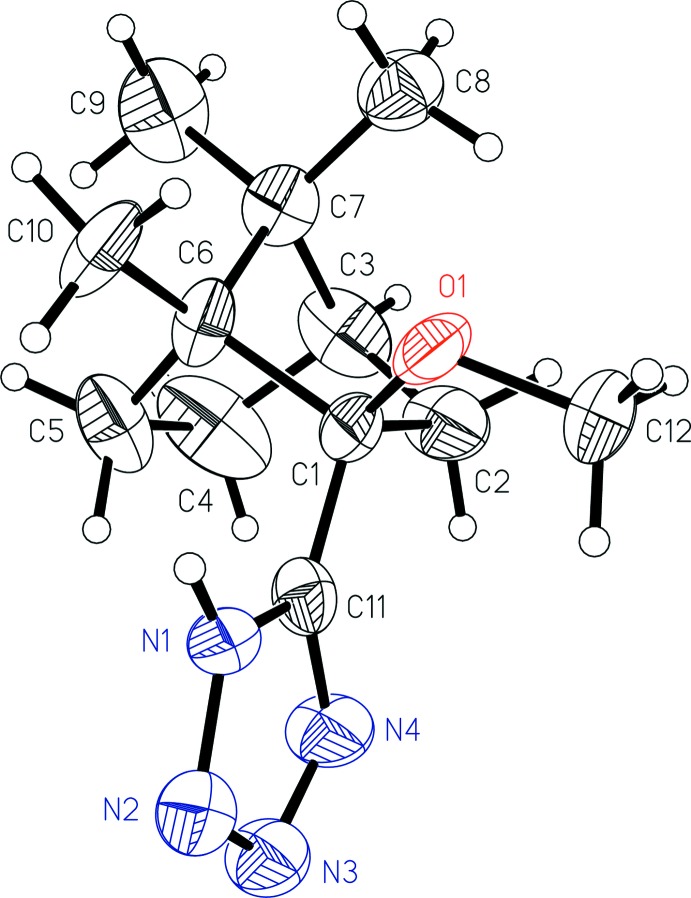
The structure of molecule A, showing the atom-labelling. Displacement ellipsoids are drawn at the 50% probability level.

**Fig. 2.**
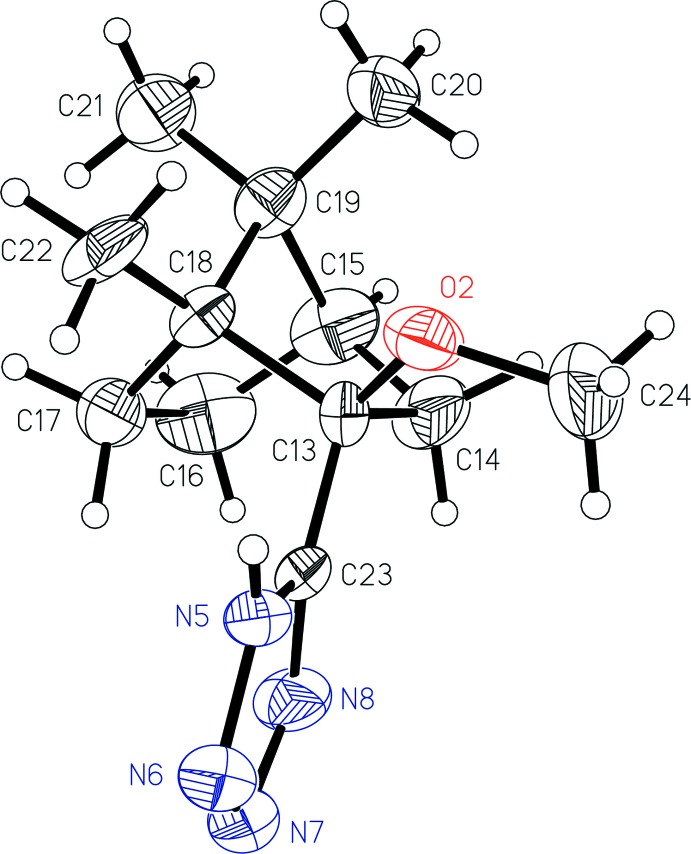
The structure of molecule B, showing the atom-labelling. Displacement ellipsoids are drawn at the 50% probability level.

**Fig. 3.**
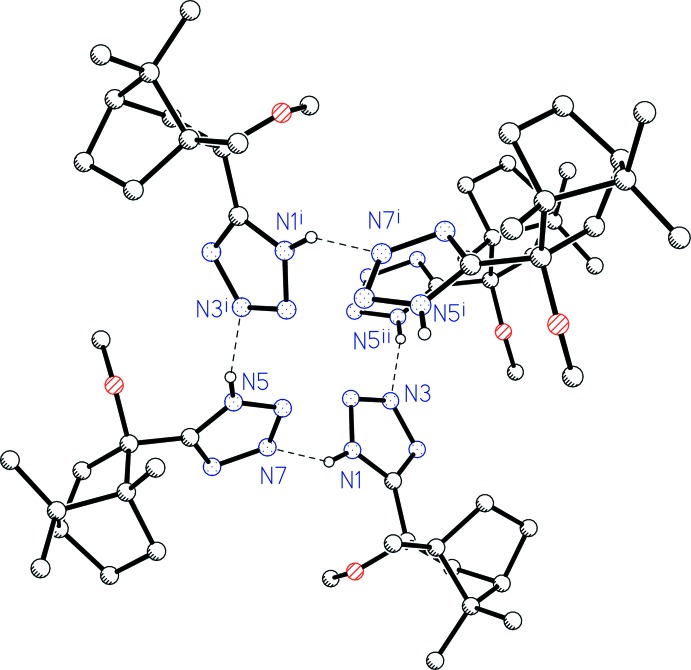
A view along the c axis of the helical structure of the title compound [the C-bound H atoms have been omitted for clarity; hydrogen bonds are shown as dashed lines; see Table 1 for details; symmetry codes: (i) -x+1/2, -y+1, z+1/2; (ii) -x+1/2, -y+1, z-1/2].

## supplementary crystallographic information

### Comment

The title compound has been prepared as a chiral catalyst for the diastereoselective synthesis of dinucleoside methylphosphonates (Schell & Engels, 1997; Schell & Engels, 1998). An initial crystal structure determination of the title compound at room temperature revealed a tetragonal unit cell with a = b = 13.452 (2) Å, c = 14.642 (2) Å and space group P4_3_2_1_2. There is one molecule in the asymmetric unit, but the atoms, especially of the methoxybornyl group show very large displacement parameters. The average *U*_eq_ values of the non-H atoms is 0.080 Å^2^ for the tetrazole group, 0.136 Å^2 ^for the bornyl group and 0.243 Å^2 ^for the methoxy group. Thus the molecule is heavily disordered. On cooling the crystal, weak incommensurate reflections were observed in the temperature range 223 - 213 K, while below 213 K an orthorhombic unit cell with space group *P*2_1_2_1_2_1_ was found. Herein, we report on the crystal structure of the orthorhombic phase measured at 194 K.

The asymmetric unit contains two independent molecules, A and B (Figs. 1 and 2). The only significant difference between the molecules is found for the torsion angles about the C_bornyl_—C_tetrazole_ bond [*e.g.* corresponding torsion angles C2—C1—C11—N4: -5.0 (7)° and C14—C13—C23—N8: -21.9 (7)°]. The methoxy group is in the *exo*-position and the tetrazole group in the *endo*-position with respect to the bicyclo[2.2.1]heptane group. The tetrazole rings are planar (r.m.s. deviations: 0.004 Å for molecule A and 0.005 Å for molecule B). The C—N bond distances in the tetrazole rings range from 1.318 (6) - 1.352 (6) Å and the N—N bond distances from 1.315 (6) - 1.353 (6) Å, thus showing a considerable degree of delocalization of the double bonds in the ring. Resonance has also been reported for tetrazole rings in other crystal structures (Ohno *et al.*, 1999).

The phase transition from the room temperature P4_3_2_1_2 structure to the low temperature *P*2_1_2_1_2_1_ structure results in the doubling of the number of molecules in the asymmetric unit from *Z*'=1 to *Z*'=2. Thus the two molecules, A and B, are expected to be related by tetragonal pseudo-symmetry. A close inspection of the fractional coordinates shows the molecules to be approximately related by the pseudo-relation: *x_B_* = -1/4 + *y_A_*, *y_B_* = 3/4 - *x_A_* and *z_B_* = 1/4 + *z_A_*. This is a symmetry element of a 4_3_ screw-axis.

In the crystal, molecules are connected by N—H···N hydrogen bonds (Table 1 and Fig. 3) to form a helix along the pseudo-4_3_ screw-axis parallel to the *c* axis direction. There are no short intermolecular contacts between neighboring helices, the shortest contact has a H···N distance of 2.71 Å. Thus the crystal habit is a [001] needle.

### Experimental

The synthesis of the title compound, starting from (+)-camphor, has been reported by Schell & Engels (1997). The final product was recrystallized from tetrachloromethane/n-hexane (1:1), resulting in colourless needles.

### Refinement

Friedel opposites were merged. C-bound H atoms were positioned geometrically and treated as riding: C—H = 0.98 - 1.00 Å, with *U*_iso_(H)=1.5*U*_eq_(C-methyl) and 1.2*U*_eq_(C) for other H atoms. N-bound H atoms were located from a difference Fourier synthesis. Their fractional coordinates were refined using a N—H bond length constraint of 0.88 (1) Å with *U*_iso_(H) = 1.2*U*_eq_(N). The crystal was found to be twinned. The twin relations are: h_twin_ = k, k_twin_ = -h and l_twin_ = l. Reflections were integrated using a large profile width. Thus the observed intensities contain the contributions of both the main and the twin reflections. Refinement of the twin fraction resulted in the value 0.231 (2).

### Crystal data

**Table:**

C_12_H_20_N_4_O	*D*_x_ = 1.208 Mg m^−^^3^
*M**_r_* = 236.32	Melting point: 446 K
Orthorhombic, *P*2_1_2_1_2_1_	Mo *K*α radiation, λ = 0.71073 Å
Hall symbol: P 2ac 2ab	Cell parameters from 76 reflections
*a* = 13.298 (3) Å	θ = 3–23°
*b* = 13.608 (4) Å	µ = 0.08 mm^−^^1^
*c* = 14.356 (3) Å	*T* = 194 K
*V* = 2597.9 (11) Å^3^	Rod, colourless
*Z* = 8	0.65 × 0.24 × 0.20 mm
*F*(000) = 1024	

### Data collection

**Table:**

Siemens SMART 1K CCD diffractometer	2259 reflections with *I* > 2σ(*I*)
Radiation source: normal-focus sealed tube	*R*_int_ = 0.109
Graphite monochromator	θ_max_ = 27.5°, θ_min_ = 2.1°
ω scans	*h* = −17→16
31860 measured reflections	*k* = −17→17
3283 independent reflections	*l* = −18→18

### Refinement

**Table:**

Refinement on *F*^2^	Primary atom site location: structure-invariant direct methods
Least-squares matrix: full	Secondary atom site location: difference Fourier map
*R*[*F*^2^ > 2σ(*F*^2^)] = 0.069	Hydrogen site location: inferred from neighbouring sites
*wR*(*F*^2^) = 0.138	H atoms treated by a mixture of independent and constrained refinement
*S* = 1.08	*w* = 1/[σ^2^(*F*_o_^2^) + (0.07*P*)^2^] where *P* = (*F*_o_^2^ + 2*F*_c_^2^)/3
3283 reflections	(Δ/σ)_max_ < 0.001
322 parameters	Δρ_max_ = 0.20 e Å^−^^3^
2 restraints	Δρ_min_ = −0.21 e Å^−^^3^

### Special details

**Table:**

Geometry. All e.s.d.'s (except the e.s.d. in the dihedral angle between two l.s. planes) are estimated using the full covariance matrix. The cell e.s.d.'s are taken into account individually in the estimation of e.s.d.'s in distances, angles and torsion angles; correlations between e.s.d.'s in cell parameters are only used when they are defined by crystal symmetry. An approximate (isotropic) treatment of cell e.s.d.'s is used for estimating e.s.d.'s involving l.s. planes.
Refinement. Refinement of *F*^2^ against ALL reflections. The weighted *R*-factor *wR* and goodness of fit *S* are based on *F*^2^, conventional *R*-factors *R* are based on *F*, with *F* set to zero for negative *F*^2^. The threshold expression of *F*^2^ > σ(*F*^2^) is used only for calculating *R*-factors(gt) *etc*. and is not relevant to the choice of reflections for refinement. *R*-factors based on *F*^2^ are statistically about twice as large as those based on *F*, and *R*- factors based on ALL data will be even larger.

### Fractional atomic coordinates and isotropic or equivalent isotropic displacement parameters (Å^2^)

**Table:**

	*x*	*y*	*z*	*U*_iso_*/*U*_eq_	
O1	0.6305 (3)	0.5594 (3)	0.3157 (2)	0.0447 (9)	
O2	0.2964 (3)	0.1694 (3)	0.6297 (2)	0.0489 (10)	
N1	0.4154 (3)	0.5552 (3)	0.3153 (3)	0.0345 (9)	
H1A	0.435 (4)	0.502 (2)	0.344 (3)	0.041*	
N2	0.3246 (3)	0.5527 (3)	0.2727 (3)	0.0466 (11)	
N3	0.3298 (3)	0.6241 (3)	0.2111 (3)	0.0471 (11)	
N4	0.4180 (4)	0.6711 (4)	0.2151 (3)	0.0509 (12)	
N5	0.3253 (3)	0.3587 (3)	0.5745 (2)	0.0309 (9)	
H5C	0.269 (2)	0.357 (4)	0.606 (3)	0.037*	
N6	0.3365 (3)	0.4372 (3)	0.5209 (3)	0.0413 (10)	
N7	0.4046 (4)	0.4110 (3)	0.4588 (3)	0.0456 (11)	
N8	0.4374 (3)	0.3188 (3)	0.4730 (3)	0.0426 (11)	
C1	0.5766 (4)	0.6494 (4)	0.3131 (3)	0.0364 (11)	
C2	0.6290 (4)	0.7309 (5)	0.2547 (4)	0.0547 (16)	
H2A	0.6921	0.7063	0.2266	0.066*	
H2B	0.5843	0.7551	0.2046	0.066*	
C3	0.6506 (5)	0.8127 (4)	0.3267 (5)	0.0589 (16)	
H3A	0.7019	0.8617	0.3059	0.071*	
C4	0.5507 (5)	0.8572 (5)	0.3526 (7)	0.085 (2)	
H4A	0.5094	0.8705	0.2966	0.102*	
H4B	0.5596	0.9191	0.3879	0.102*	
C5	0.5031 (4)	0.7775 (4)	0.4133 (5)	0.0591 (17)	
H5A	0.4908	0.8020	0.4772	0.071*	
H5B	0.4386	0.7548	0.3864	0.071*	
C6	0.5800 (4)	0.6954 (4)	0.4136 (3)	0.0419 (12)	
C7	0.6814 (4)	0.7541 (4)	0.4126 (4)	0.0443 (13)	
C8	0.7776 (4)	0.6940 (4)	0.4010 (4)	0.0526 (14)	
H8A	0.8355	0.7384	0.3971	0.079*	
H8B	0.7733	0.6547	0.3439	0.079*	
H8C	0.7858	0.6502	0.4547	0.079*	
C9	0.6968 (5)	0.8181 (5)	0.5005 (5)	0.0683 (19)	
H9A	0.7494	0.8670	0.4886	0.102*	
H9B	0.7170	0.7762	0.5528	0.102*	
H9C	0.6338	0.8517	0.5159	0.102*	
C10	0.5666 (5)	0.6200 (5)	0.4921 (3)	0.0581 (18)	
H10A	0.5834	0.6506	0.5519	0.087*	
H10B	0.6112	0.5639	0.4812	0.087*	
H10C	0.4966	0.5975	0.4933	0.087*	
C11	0.4718 (4)	0.6263 (4)	0.2813 (3)	0.0358 (11)	
C12	0.6516 (5)	0.5179 (5)	0.2245 (4)	0.0641 (17)	
H12A	0.5905	0.5196	0.1864	0.096*	
H12B	0.6740	0.4497	0.2316	0.096*	
H12C	0.7045	0.5563	0.1941	0.096*	
C13	0.3936 (3)	0.1902 (4)	0.5947 (3)	0.0337 (11)	
C14	0.4411 (5)	0.1082 (4)	0.5341 (4)	0.0549 (16)	
H14A	0.3939	0.0525	0.5265	0.066*	
H14B	0.4593	0.1337	0.4717	0.066*	
C15	0.5344 (5)	0.0766 (4)	0.5875 (4)	0.0558 (16)	
H15A	0.5603	0.0102	0.5696	0.067*	
C16	0.6124 (5)	0.1578 (5)	0.5818 (4)	0.0659 (18)	
H16A	0.6209	0.1811	0.5169	0.079*	
H16B	0.6782	0.1355	0.6060	0.079*	
C17	0.5673 (4)	0.2382 (4)	0.6436 (4)	0.0456 (13)	
H17A	0.5544	0.2988	0.6074	0.055*	
H17B	0.6127	0.2539	0.6962	0.055*	
C18	0.4685 (3)	0.1927 (4)	0.6786 (3)	0.0317 (10)	
C19	0.4982 (4)	0.0841 (4)	0.6911 (3)	0.0399 (12)	
C20	0.4155 (5)	0.0138 (4)	0.7167 (4)	0.0573 (15)	
H20A	0.3924	0.0277	0.7802	0.086*	
H20B	0.4408	−0.0538	0.7134	0.086*	
H20C	0.3592	0.0216	0.6732	0.086*	
C21	0.5845 (5)	0.0671 (5)	0.7623 (4)	0.0567 (15)	
H21A	0.5573	0.0695	0.8257	0.085*	
H21B	0.6355	0.1185	0.7548	0.085*	
H21C	0.6151	0.0027	0.7512	0.085*	
C22	0.4253 (5)	0.2447 (4)	0.7636 (3)	0.0468 (14)	
H22A	0.4756	0.2455	0.8135	0.070*	
H22B	0.3652	0.2097	0.7851	0.070*	
H22C	0.4073	0.3123	0.7470	0.070*	
C23	0.3845 (3)	0.2864 (4)	0.5473 (3)	0.0300 (10)	
C24	0.2217 (5)	0.1422 (5)	0.5634 (5)	0.080 (2)	
H24A	0.2365	0.1735	0.5034	0.121*	
H24B	0.1555	0.1639	0.5853	0.121*	
H24C	0.2216	0.0707	0.5557	0.121*	

### Atomic displacement parameters (Å^2^)

**Table:**

	*U*^11^	*U*^22^	*U*^33^	*U*^12^	*U*^13^	*U*^23^
O1	0.056 (2)	0.054 (2)	0.0242 (15)	0.0130 (18)	−0.0103 (15)	−0.0055 (16)
O2	0.044 (2)	0.051 (2)	0.052 (2)	−0.0146 (18)	−0.0080 (17)	0.0216 (18)
N1	0.035 (2)	0.035 (2)	0.033 (2)	0.002 (2)	−0.0031 (18)	0.0015 (18)
N2	0.046 (3)	0.053 (3)	0.041 (2)	−0.002 (2)	0.002 (2)	0.001 (2)
N3	0.046 (3)	0.053 (3)	0.042 (2)	0.000 (2)	−0.004 (2)	0.010 (2)
N4	0.049 (3)	0.066 (3)	0.038 (2)	0.001 (2)	−0.008 (2)	0.025 (2)
N5	0.035 (2)	0.029 (2)	0.0285 (19)	−0.0009 (19)	−0.0028 (17)	0.0040 (18)
N6	0.052 (3)	0.033 (2)	0.039 (2)	−0.002 (2)	−0.005 (2)	0.007 (2)
N7	0.065 (3)	0.042 (3)	0.029 (2)	−0.012 (2)	−0.003 (2)	0.0046 (19)
N8	0.060 (3)	0.044 (3)	0.0239 (19)	0.002 (2)	0.0066 (19)	0.0010 (19)
C1	0.039 (3)	0.046 (3)	0.024 (2)	0.003 (2)	0.002 (2)	0.006 (2)
C2	0.044 (3)	0.070 (4)	0.049 (3)	−0.001 (3)	0.000 (3)	0.029 (3)
C3	0.050 (4)	0.040 (3)	0.087 (4)	−0.005 (3)	0.006 (3)	0.020 (3)
C4	0.058 (4)	0.047 (4)	0.150 (7)	0.003 (3)	0.005 (4)	0.012 (4)
C5	0.044 (3)	0.051 (4)	0.082 (4)	−0.006 (3)	0.020 (3)	−0.011 (3)
C6	0.058 (3)	0.038 (3)	0.030 (2)	−0.014 (3)	0.011 (2)	−0.006 (2)
C7	0.053 (3)	0.034 (3)	0.046 (3)	−0.007 (3)	0.001 (3)	−0.001 (2)
C8	0.061 (4)	0.047 (3)	0.050 (3)	0.004 (3)	−0.005 (3)	0.000 (3)
C9	0.072 (4)	0.047 (4)	0.086 (5)	−0.014 (3)	0.009 (4)	−0.026 (3)
C10	0.087 (5)	0.067 (4)	0.021 (2)	−0.022 (3)	−0.001 (3)	−0.002 (2)
C11	0.036 (3)	0.037 (3)	0.034 (2)	−0.001 (2)	0.008 (2)	0.004 (2)
C12	0.062 (4)	0.088 (5)	0.042 (3)	0.010 (4)	0.001 (3)	−0.029 (3)
C13	0.036 (3)	0.034 (3)	0.032 (2)	−0.011 (2)	−0.008 (2)	0.000 (2)
C14	0.091 (5)	0.040 (3)	0.034 (3)	0.008 (3)	−0.008 (3)	−0.008 (2)
C15	0.078 (4)	0.048 (3)	0.042 (3)	0.026 (3)	0.007 (3)	−0.009 (3)
C16	0.051 (4)	0.087 (5)	0.060 (3)	0.013 (4)	0.021 (3)	0.009 (4)
C17	0.041 (3)	0.047 (3)	0.049 (3)	−0.005 (3)	−0.008 (2)	0.007 (2)
C18	0.033 (2)	0.038 (3)	0.025 (2)	0.005 (2)	−0.0033 (19)	0.001 (2)
C19	0.038 (3)	0.043 (3)	0.038 (3)	0.010 (2)	−0.003 (2)	−0.001 (2)
C20	0.066 (4)	0.046 (3)	0.061 (3)	−0.014 (3)	−0.021 (3)	0.017 (3)
C21	0.053 (3)	0.053 (4)	0.063 (4)	0.003 (3)	−0.012 (3)	0.008 (3)
C22	0.069 (4)	0.053 (3)	0.018 (2)	0.017 (3)	−0.004 (2)	−0.002 (2)
C23	0.038 (3)	0.035 (3)	0.0172 (19)	−0.005 (2)	−0.0053 (18)	0.0019 (19)
C24	0.071 (5)	0.072 (5)	0.099 (5)	−0.034 (4)	−0.044 (4)	0.023 (4)

### Geometric parameters (Å, º)

**Table:**

O1—C1	1.420 (6)	C9—H9B	0.9800
O1—C12	1.453 (6)	C9—H9C	0.9800
O2—C13	1.416 (6)	C10—H10A	0.9800
O2—C24	1.425 (7)	C10—H10B	0.9800
N1—C11	1.318 (6)	C10—H10C	0.9800
N1—N2	1.353 (6)	C12—H12A	0.9800
N1—H1A	0.877 (10)	C12—H12B	0.9800
N2—N3	1.315 (6)	C12—H12C	0.9800
N3—N4	1.337 (6)	C13—C23	1.479 (7)
N4—C11	1.337 (6)	C13—C14	1.549 (7)
N5—C23	1.320 (6)	C13—C18	1.564 (6)
N5—N6	1.325 (5)	C14—C15	1.520 (8)
N5—H5C	0.879 (10)	C14—H14A	0.9900
N6—N7	1.319 (6)	C14—H14B	0.9900
N7—N8	1.344 (6)	C15—C16	1.518 (9)
N8—C23	1.352 (6)	C15—C19	1.567 (7)
C1—C11	1.499 (7)	C15—H15A	1.0000
C1—C2	1.555 (7)	C16—C17	1.530 (8)
C1—C6	1.574 (7)	C16—H16A	0.9900
C2—C3	1.546 (9)	C16—H16B	0.9900
C2—H2A	0.9900	C17—C18	1.537 (7)
C2—H2B	0.9900	C17—H17A	0.9900
C3—C4	1.507 (9)	C17—H17B	0.9900
C3—C7	1.524 (8)	C18—C22	1.523 (7)
C3—H3A	1.0000	C18—C19	1.540 (7)
C4—C5	1.529 (9)	C19—C20	1.504 (7)
C4—H4A	0.9900	C19—C21	1.554 (7)
C4—H4B	0.9900	C20—H20A	0.9800
C5—C6	1.515 (8)	C20—H20B	0.9800
C5—H5A	0.9900	C20—H20C	0.9800
C5—H5B	0.9900	C21—H21A	0.9800
C6—C10	1.534 (7)	C21—H21B	0.9800
C6—C7	1.567 (7)	C21—H21C	0.9800
C7—C8	1.527 (8)	C22—H22A	0.9800
C7—C9	1.548 (8)	C22—H22B	0.9800
C8—H8A	0.9800	C22—H22C	0.9800
C8—H8B	0.9800	C24—H24A	0.9800
C8—H8C	0.9800	C24—H24B	0.9800
C9—H9A	0.9800	C24—H24C	0.9800
			
C1—O1—C12	114.1 (4)	N4—C11—C1	128.2 (4)
C13—O2—C24	116.8 (4)	O1—C12—H12A	109.5
C11—N1—N2	111.1 (4)	O1—C12—H12B	109.5
C11—N1—H1A	128 (3)	H12A—C12—H12B	109.5
N2—N1—H1A	117 (3)	O1—C12—H12C	109.5
N3—N2—N1	103.8 (4)	H12A—C12—H12C	109.5
N2—N3—N4	111.8 (4)	H12B—C12—H12C	109.5
N3—N4—C11	106.3 (4)	O2—C13—C23	105.4 (4)
C23—N5—N6	111.3 (4)	O2—C13—C14	115.3 (4)
C23—N5—H5C	130 (3)	C23—C13—C14	114.4 (4)
N6—N5—H5C	114 (3)	O2—C13—C18	108.2 (4)
N7—N6—N5	104.5 (4)	C23—C13—C18	112.8 (4)
N6—N7—N8	111.9 (4)	C14—C13—C18	100.9 (4)
N7—N8—C23	104.8 (4)	C15—C14—C13	104.7 (4)
O1—C1—C11	107.2 (4)	C15—C14—H14A	110.8
O1—C1—C2	113.8 (4)	C13—C14—H14A	110.8
C11—C1—C2	113.7 (4)	C15—C14—H14B	110.8
O1—C1—C6	107.7 (4)	C13—C14—H14B	110.8
C11—C1—C6	113.0 (4)	H14A—C14—H14B	108.9
C2—C1—C6	101.3 (4)	C16—C15—C14	108.9 (5)
C3—C2—C1	103.7 (4)	C16—C15—C19	102.3 (5)
C3—C2—H2A	111.0	C14—C15—C19	102.1 (4)
C1—C2—H2A	111.0	C16—C15—H15A	114.1
C3—C2—H2B	111.0	C14—C15—H15A	114.1
C1—C2—H2B	111.0	C19—C15—H15A	114.1
H2A—C2—H2B	109.0	C15—C16—C17	102.8 (4)
C4—C3—C7	104.4 (6)	C15—C16—H16A	111.2
C4—C3—C2	106.9 (6)	C17—C16—H16A	111.2
C7—C3—C2	102.3 (4)	C15—C16—H16B	111.2
C4—C3—H3A	114.0	C17—C16—H16B	111.2
C7—C3—H3A	114.0	H16A—C16—H16B	109.1
C2—C3—H3A	114.0	C16—C17—C18	103.7 (4)
C3—C4—C5	102.7 (5)	C16—C17—H17A	111.0
C3—C4—H4A	111.2	C18—C17—H17A	111.0
C5—C4—H4A	111.2	C16—C17—H17B	111.0
C3—C4—H4B	111.2	C18—C17—H17B	111.0
C5—C4—H4B	111.2	H17A—C17—H17B	109.0
H4A—C4—H4B	109.1	C22—C18—C17	113.4 (4)
C6—C5—C4	104.2 (5)	C22—C18—C19	116.7 (4)
C6—C5—H5A	110.9	C17—C18—C19	101.8 (4)
C4—C5—H5A	110.9	C22—C18—C13	112.8 (4)
C6—C5—H5B	110.9	C17—C18—C13	107.5 (4)
C4—C5—H5B	110.9	C19—C18—C13	103.5 (4)
H5A—C5—H5B	108.9	C20—C19—C18	116.8 (4)
C5—C6—C10	114.6 (5)	C20—C19—C21	106.6 (4)
C5—C6—C7	101.9 (4)	C18—C19—C21	114.1 (4)
C10—C6—C7	116.6 (5)	C20—C19—C15	114.5 (5)
C5—C6—C1	105.7 (5)	C18—C19—C15	91.8 (4)
C10—C6—C1	113.9 (4)	C21—C19—C15	112.8 (4)
C7—C6—C1	102.7 (4)	C19—C20—H20A	109.5
C3—C7—C8	114.7 (5)	C19—C20—H20B	109.5
C3—C7—C9	113.6 (4)	H20A—C20—H20B	109.5
C8—C7—C9	106.2 (5)	C19—C20—H20C	109.5
C3—C7—C6	92.5 (4)	H20A—C20—H20C	109.5
C8—C7—C6	116.6 (4)	H20B—C20—H20C	109.5
C9—C7—C6	113.2 (5)	C19—C21—H21A	109.5
C7—C8—H8A	109.5	C19—C21—H21B	109.5
C7—C8—H8B	109.5	H21A—C21—H21B	109.5
H8A—C8—H8B	109.5	C19—C21—H21C	109.5
C7—C8—H8C	109.5	H21A—C21—H21C	109.5
H8A—C8—H8C	109.5	H21B—C21—H21C	109.5
H8B—C8—H8C	109.5	C18—C22—H22A	109.5
C7—C9—H9A	109.5	C18—C22—H22B	109.5
C7—C9—H9B	109.5	H22A—C22—H22B	109.5
H9A—C9—H9B	109.5	C18—C22—H22C	109.5
C7—C9—H9C	109.5	H22A—C22—H22C	109.5
H9A—C9—H9C	109.5	H22B—C22—H22C	109.5
H9B—C9—H9C	109.5	N5—C23—N8	107.5 (4)
C6—C10—H10A	109.5	N5—C23—C13	125.0 (4)
C6—C10—H10B	109.5	N8—C23—C13	127.5 (5)
H10A—C10—H10B	109.5	O2—C24—H24A	109.5
C6—C10—H10C	109.5	O2—C24—H24B	109.5
H10A—C10—H10C	109.5	H24A—C24—H24B	109.5
H10B—C10—H10C	109.5	O2—C24—H24C	109.5
N1—C11—N4	107.0 (4)	H24A—C24—H24C	109.5
N1—C11—C1	124.8 (4)	H24B—C24—H24C	109.5
			
C11—N1—N2—N3	0.9 (5)	O1—C1—C11—N4	−131.7 (5)
N1—N2—N3—N4	−1.2 (5)	C2—C1—C11—N4	−5.0 (7)
N2—N3—N4—C11	1.1 (6)	C6—C1—C11—N4	109.8 (6)
C23—N5—N6—N7	0.4 (5)	C24—O2—C13—C23	72.8 (5)
N5—N6—N7—N8	−1.1 (5)	C24—O2—C13—C14	−54.3 (6)
N6—N7—N8—C23	1.3 (5)	C24—O2—C13—C18	−166.3 (5)
C12—O1—C1—C11	70.1 (5)	O2—C13—C14—C15	−117.3 (5)
C12—O1—C1—C2	−56.6 (6)	C23—C13—C14—C15	120.3 (5)
C12—O1—C1—C6	−168.1 (4)	C18—C13—C14—C15	−1.1 (5)
O1—C1—C2—C3	−117.8 (5)	C13—C14—C15—C16	−71.0 (5)
C11—C1—C2—C3	119.0 (5)	C13—C14—C15—C19	36.7 (6)
C6—C1—C2—C3	−2.5 (6)	C14—C15—C16—C17	70.9 (6)
C1—C2—C3—C4	−70.5 (6)	C19—C15—C16—C17	−36.6 (5)
C1—C2—C3—C7	38.9 (6)	C15—C16—C17—C18	0.6 (5)
C7—C3—C4—C5	−34.8 (7)	C16—C17—C18—C22	162.5 (4)
C2—C3—C4—C5	73.1 (7)	C16—C17—C18—C19	36.3 (5)
C3—C4—C5—C6	−1.2 (7)	C16—C17—C18—C13	−72.1 (5)
C4—C5—C6—C10	162.2 (5)	O2—C13—C18—C22	−41.4 (6)
C4—C5—C6—C7	35.4 (6)	C23—C13—C18—C22	74.7 (5)
C4—C5—C6—C1	−71.6 (6)	C14—C13—C18—C22	−162.8 (4)
O1—C1—C6—C5	−167.4 (4)	O2—C13—C18—C17	−167.2 (4)
C11—C1—C6—C5	−49.1 (5)	C23—C13—C18—C17	−51.0 (5)
C2—C1—C6—C5	72.9 (5)	C14—C13—C18—C17	71.4 (5)
O1—C1—C6—C10	−40.7 (6)	O2—C13—C18—C19	85.6 (4)
C11—C1—C6—C10	77.5 (6)	C23—C13—C18—C19	−158.3 (4)
C2—C1—C6—C10	−160.5 (5)	C14—C13—C18—C19	−35.8 (5)
O1—C1—C6—C7	86.2 (4)	C22—C18—C19—C20	61.6 (6)
C11—C1—C6—C7	−155.5 (4)	C17—C18—C19—C20	−174.3 (4)
C2—C1—C6—C7	−33.5 (5)	C13—C18—C19—C20	−62.9 (5)
C4—C3—C7—C8	175.1 (5)	C22—C18—C19—C21	−63.6 (6)
C2—C3—C7—C8	63.8 (6)	C17—C18—C19—C21	60.4 (5)
C4—C3—C7—C9	−62.5 (7)	C13—C18—C19—C21	171.9 (4)
C2—C3—C7—C9	−173.8 (5)	C22—C18—C19—C15	−179.6 (5)
C4—C3—C7—C6	54.2 (5)	C17—C18—C19—C15	−55.6 (4)
C2—C3—C7—C6	−57.1 (5)	C13—C18—C19—C15	55.9 (4)
C5—C6—C7—C3	−53.7 (5)	C16—C15—C19—C20	177.4 (5)
C10—C6—C7—C3	−179.2 (5)	C14—C15—C19—C20	64.7 (6)
C1—C6—C7—C3	55.6 (5)	C16—C15—C19—C18	56.7 (5)
C5—C6—C7—C8	−173.1 (5)	C14—C15—C19—C18	−56.0 (5)
C10—C6—C7—C8	61.5 (6)	C16—C15—C19—C21	−60.5 (6)
C1—C6—C7—C8	−63.7 (6)	C14—C15—C19—C21	−173.2 (5)
C5—C6—C7—C9	63.3 (6)	N6—N5—C23—N8	0.4 (5)
C10—C6—C7—C9	−62.2 (6)	N6—N5—C23—C13	177.2 (4)
C1—C6—C7—C9	172.6 (5)	N7—N8—C23—N5	−1.0 (5)
N2—N1—C11—N4	−0.3 (5)	N7—N8—C23—C13	−177.7 (4)
N2—N1—C11—C1	179.9 (4)	O2—C13—C23—N5	34.3 (6)
N3—N4—C11—N1	−0.4 (5)	C14—C13—C23—N5	162.0 (4)
N3—N4—C11—C1	179.4 (5)	C18—C13—C23—N5	−83.5 (6)
O1—C1—C11—N1	48.0 (6)	O2—C13—C23—N8	−149.5 (4)
C2—C1—C11—N1	174.8 (5)	C14—C13—C23—N8	−21.9 (7)
C6—C1—C11—N1	−70.4 (6)	C18—C13—C23—N8	92.7 (5)

### Hydrogen-bond geometry (Å, º)

**Table:**

*D*—H···*A*	*D*—H	H···*A*	*D*···*A*	*D*—H···*A*
N1—H1*A*···N7	0.88 (1)	2.09 (3)	2.850 (6)	144 (5)
N5—H5*C*···N3^i^	0.88 (1)	2.02 (2)	2.857 (6)	160 (5)

Symmetry code: (i) −*x*+1/2, −*y*+1, *z*+1/2.
